# The Management of Left Subclavian Artery in Aortic Open and Endovascular Surgery

**DOI:** 10.3390/jcdd13090468

**Published:** 2026-09-16

**Authors:** Federico Giorgi, Nicola Troisi, Laura Besola, Danilo Ruggiero, Giacomo Ravenni, Michele Celiento, Andrea Colli

**Affiliations:** 1 Cardiac Surgery Division, Department of Surgical, Medical and Molecular Pathology and Critical Care, University of Pisa, 56124 Pisa, Italy; giorgifederico97@gmail.com (F.G.); laura.besola@hotmail.it (L.B.); danilo.ruggiero83.dr@gmail.com (D.R.); giacomoravenni@gmail.com (G.R.); miceliento@gmail.com (M.C.); 2 Vascular Surgery Unit, Department of Translational Research and New Technologies in Medicine and Surgery, University of Pisa, 56124 Pisa, Italy; nicola.troisi@unipi.it

**Keywords:** aortic arch, left subclavian artery, open surgery, endovascular, hybrid

## Abstract

Management of the left subclavian artery (LSA) during aortic arch surgery is complex due to its deep anatomical location and potential pathological alterations, posing risks such as stroke, spinal cord injury, and recurrent laryngeal nerve damage. Revascularization of the LSA is critical in various clinical scenarios to maintain adequate perfusion to the vertebral arteries, spinal cord, and upper limb, especially in patients with anatomical variations, prior bypass surgeries, or increased risk of ischemia. Coverage of the LSA during thoracic endovascular repair (TEVAR) without revascularization significantly increases stroke and spinal cord ischemia rates. We provided a narrative review, evaluating contemporary open and endovascular options for the treatment of the aortic arch, and consequently of the LSA. Frozen elephant trunk (FET), endovascular strategies (branched and fenestrated TEVAR), but also emerging hybrid devices, are all valuable treatments with respective strengths and limitations to be understood to obtain the best patient-prosthesis match. Individualized treatment selection based on pathology, urgency, and patient factors is essential to optimize outcomes. This evolving paradigm integrates open and endovascular techniques to enhance patient care in complex aortic arch disease.

## 1. Introduction

Management of the left subclavian artery (LSA) during aortic arch surgery presents several challenges, including preserving distal perfusion, achieving hemostasis and preventing stroke and spinal cord injury in the posterior circulation. The most common challenge remains its deep location in the chest, often exacerbated by posterior and apical displacement due to an arch aneurysm [1,2]. An additional challenge arises when the LSA itself is aneurysmal, dissected, calcified, fragile or adherent from previous surgery; direct manipulation of the LSA is associated with a higher risk of injury to the recurrent laryngeal nerve [3]. During thoracic endovascular aortic repair (TEVAR), LSA management may likewise be challenging because of these anatomical considerations.

## 2. Why LSA Must Be Revascularized

Revascularization of the LSA may be indicated in several clinical settings. First, if the right subclavian artery is absent, has diminished flow, or the patient has a dominant left vertebral artery, the LSA is essential to ensure adequate perfusion of the vertebral arteries. Second, LSA perfusion should be preserved when a previous surgical bypass depends on it, such as a left internal mammary artery (LIMA) coronary bypass graft or a left axillofemoral bypass. Third, preservation of LSA flow may be particularly relevant in left-handed patients to maintain optimal function of the dominant arm. Additionally, in patients at higher risk for spinal cord injury, such as in the case of previous descending thoracic or abdominal aortic replacement, LSA ligation or coverage may compromise spinal cord perfusion and increase the risk of ischemia. Moreover, many patients presenting with aortic arch pathologies have pan-aortic disease and will often require multiple aortic procedures in their lifetime; thus, preservation of perfusion to the spinal cord is paramount in preventing catastrophic paraplegia.

Covering the LSA has implications that extend beyond reducing blood flow to the left arm. Left arm ischemia is a rare complication of LSA coverage owing to the rich collateral network around the upper extremity, and flow to the left arm is usually maintained by compensatory blood flow through the circle of Willis in the brain, with reversal of flow down the left vertebral artery into the arm. A more clinically significant concern is spinal cord ischemia, which may result in paraparesis or even permanent paraplegia. The LSA is an important collateral route of blood supply to the spinal cord, which receives segmental blood supply from the descending branches of the thoracic aorta, such as the intercostal arteries [4].

During TEVAR, the segmental feeding arteries to the spinal cord are routinely covered and excluded. Stroke is a significant additional concern, with patients undergoing TEVAR without routine LSA revascularization showing substantially higher rates of stroke [14.3%] than patients undergoing TEVAR with LSA revascularization (1.9%) [5].

Revascularization techniques include reimplantation within an island patch or direct anastomosis, stenting, bypass, transposition or a hybrid approach.

Another important but little-discussed scenario is the management of the left vertebral artery arising directly from the aorta. With a prevalence of 4% to 7%, this is the second most common anatomical variation in the aortic arch [6].

Meta-analyses consistently show that performing LSA revascularization, when coverage is required, reduces the risk of stroke [particularly in the posterior circulation], spinal cord ischemia [SCI], and upper-limb ischemia compared with no revascularization [7,8,9].

The most recent European guidelines recommend frozen elephant trunk (FET) for type A aortic dissection with arch tear or aneurysm (IIb) and TEVAR for complicated type B aortic dissection (Ib). Open arch replacement should be considered for type B dissections unsuitable for TEVAR (IIa). Regarding LSA revascularization during arch repair, a distinction between TEVAR and FET is noted. The guidelines recommend, in class Ib, revascularization of the LSA in any TEVAR involving zone 2, whereas no equivalent recommendation is provided for FET [10,11].

The clinical setting strongly influences the range of available treatments. In acute type A dissection, emergent surgery is generally required, whereas type B dissection and, particularly, chronic thoracic aneurysms allow a broader range of elective treatment options. Accordingly, off-the-shelf devices are particularly valuable in urgent or emergent settings, whereas custom-made devices, despite longer manufacturing times, represent useful alternatives in elective cases.

## 3. Methods

This narrative review was conducted to provide a comprehensive overview of current and emerging strategies for LSA management and revascularization in the setting of aortic arch surgery and TEVAR, with particular emphasis on technical aspects, clinical applicability, advantages, limitations, and evolving technologies.

A comprehensive literature search was performed using PubMed/MEDLINE, Scopus, and Web of Science to identify relevant publications addressing LSA management during open, hybrid, and endovascular treatment of aortic arch disease. The search included studies published from January 1990 to August 2026, with particular attention to contemporary techniques and devices. The search strategy combined terms related to the LSA and aortic arch interventions, including: “left subclavian artery,” “LSA revascularization,” “aortic arch surgery,” “aortic arch replacement,” “frozen elephant trunk,”, “thoracic endovascular aortic repair,” “TEVAR,” “carotid-subclavian bypass,”, “hybrid aortic arch repair,” “fenestrated TEVAR,” “branched TEVAR,” “in situ fenestration,” “chimney technique,” and “supra-aortic vessel revascularization.” Reference lists of relevant articles and reviews were additionally screened to identify further pertinent publications.

Eligible publications included original clinical studies, prospective and retrospective observational studies, clinical trials, registries, technical reports and case series describing established or innovative approaches to LSA preservation or revascularization. Relevant systematic reviews, meta-analyses, consensus documents, and international guidelines were also considered to provide contextual evidence and support current recommendations. Case reports were considered selectively when describing novel techniques or devices for which larger clinical series were not yet available. Articles not written in English, publications without sufficient technical or clinical information, and studies not directly addressing LSA management in the context of thoracic or aortic arch interventions were excluded.

Given the rapid evolution of hybrid and endovascular technologies, additional searches of ClinicalTrials.gov and manufacturers’ publicly available technical information were performed to identify ongoing clinical trials and recently introduced devices for which peer-reviewed clinical evidence remains limited. Information derived from these sources was used primarily to describe device characteristics and developmental status rather than to establish comparative clinical efficacy.

The retrieved evidence was organized according to the principal therapeutic strategies: open surgical approaches, including carotid-to-subclavian bypass and frozen elephant trunk techniques; hybrid approaches, including fenestrated FET, stented-branch and bridging-stent techniques; and endovascular approaches, including chimney techniques, in situ fenestration, fenestrated TEVAR, and branched TEVAR. For each strategy, technical characteristics, indications, anatomical considerations, reported clinical outcomes, strengths, limitations, and applicability in elective, urgent, and emergent settings were narratively synthesized.

Because of the narrative nature of the review and the substantial heterogeneity in study design, patient populations, aortic pathology, devices, procedural techniques, and reported outcomes, no formal quantitative meta-analysis or standardized risk-of-bias assessment was performed. Evidence was interpreted qualitatively, with preference given to contemporary studies, larger clinical series, comparative studies, multicenter experiences, systematic reviews, and studies providing longer-term follow-up. Particular attention was also given to the distinction between established techniques supported by mature clinical evidence and emerging technologies for which evidence is currently limited to early feasibility studies or small clinical series.

## 4. Open Aortic Surgery

### 4.1. Carotid-to-Subclavian Bypass (CSB)

CSB is performed with a transverse supraclavicular incision [6–8 cm] made across the heads of the sternocleidomastoid. The platysma is divided, the internal jugular vein is identified, and the carotid sheath is opened while protecting the vagus nerve. The graft is sewn end-to-side to the left common carotid artery (LCCA) on its lateral aspect. The graft is tunneled posterior to the clavicle and anastomosed end-to-side to the subclavian artery.

It is widely performed for revascularization of the LSA, likely owing to familiarity with the exposure and the relative technical simplicity of the procedure.

CSB is associated with excellent long-term durability and outcomes and is generally accepted as one of the standard options for LSA revascularization [12]. Caution is warranted in patients with previous radiation to the cervical region, tracheostomy, modified radical neck dissection or previous neck surgery, unstable cervical spine, severe obesity or stiff neck precluding adequate surgical exposure [13].

When placing a straight TEVAR prosthesis landing in zone 2 (and thus covering the LSA), one of the most commonly adopted techniques to preserve the LSA is the CSB. It can also represent a valuable alternative in cases of a displaced LSA during FET, to simplify supra-aortic vessel management. Thus, it represents a “bridging strategy” helpful for both open and endovascular surgery.

Strenghts

Excellent long-term patency and relative low-risk procedure.

Limitations

It usually represents one stage of a two-stage procedure (CSB and TEVAR), although a one-stage approach may also be considered. In urgent or emergent settings, CSB is generally not the first-line option. The left cerebral circulation then depends on a single inflow vessel (the LCCA), which may confer an additional stroke risk, although this risk is low in contemporary series [14].

### 4.2. Frozen Elephant Trunk (FET)

The advent of the FET technique revolutionized the treatment of aortic arch diseases. FET requires lower-body circulatory arrest, usually combined with antegrade cerebral perfusion and mild-to-deep hypothermia [15]; even normothermic FET has been described with encouraging results [16]. Complications associated with this surgery include SCI [paraparesis/paraplegia], neurological events, intraoperative mortality, renal/gastrointestinal complications, and hemorrhagic and thrombotic risks associated with longer CPB times [17].

Evita Open-NEO (Artivion, Kennesaw, GA, USA) and Thoraflex Hybrid (Terumo Aortic, Sunrise, FL, USA), introduced in the first decade of the 2000s, are now the most commonly used platforms worldwide. Evita Open-NEO is characterized by longer stent availability (up to 180 mm) and a more balanced sizing, useful in cases of a large aortic root/ascending aorta and a proportionate aortic arch. It is available in a typical one-branch (straight) origin from the surgical graft of vascular branches, as well as branched (2 branches) or trifurcated (3 branches). In daily surgical practice the porosity of surgical graft [not gelatine-coated] requires longer hemostasis to obtain an optimal sealing. Thoraflex prosthesis is designed with a Gealweave dacron graft, optimal for sewing and a faster sealing (reducing hemostasis time). Available in plexus (3 branches + auxiliary branch) and Ante-flo/island conformation. Sizing is conceived with one size difference from proximal to distal (24/26, 28/30), not ideal in case of root larger than aortic arch. Stent from 100 to 150 mm is available. Cronus (MicroPort Endovascular MedTech, Shanghai, China) with a polyester graft and nitinol stented graft similar to Evita in sizing and Frozenix J graft (Japan Lifeline, Tokyo, Japan), with a dacron graft and nitinol self-expandable stented graft, are limited in use to China and Japan, respectively. Available clinical data have shown favorable outcomes consistent with previously published experience [18,19,20,21].

The use of an anastomotic collar compensates for any graft-to-aorta discrepancy in diameter and facilitates a more proximal anastomosis (just distal to the LCCA instead of distal to the LSA); in this approach, the LSA may be bypassed or left in its native state without revascularization (with all related risks) [22]. LSA management can be achieved in different ways.

Direct LSA anastomosis to the vascular branch of the FET prosthesis is conceptually the most straightforward approach. All available FET prostheses have dedicated vascular branches for direct anastomosis, with differences in terms of single-branch origin from main body and orientation of branches. Over recent decades, this approach has become established as a standard of care with evident advantages compared with the original elephant trunk and large registries confirming acceptable mortality and morbidity among patients with complex aortic pathologies [23]. However, posterior displacement of the LSA in arch aneurysms or dissections, as well as a distal LSA origin, can make direct anastomosis quite demanding in many cases.

An extra-anatomical bypass with an additional vascular branch of the FET prosthesis, to the surgically isolated subclavian artery represents a useful alternative, although the deep and technically demanding LSA-to-graft anastomosis may complicate hemostasis [24]. Surgical isolation of the LSA, routinely done in cardiac surgery, may risk brachial plexus injury and can be particularly challenging in patients with obesity. In this case, the native LSA can be closed directly during surgery or, if management is difficult, closed percutaneously a few days after surgery.

**Figure 1:**
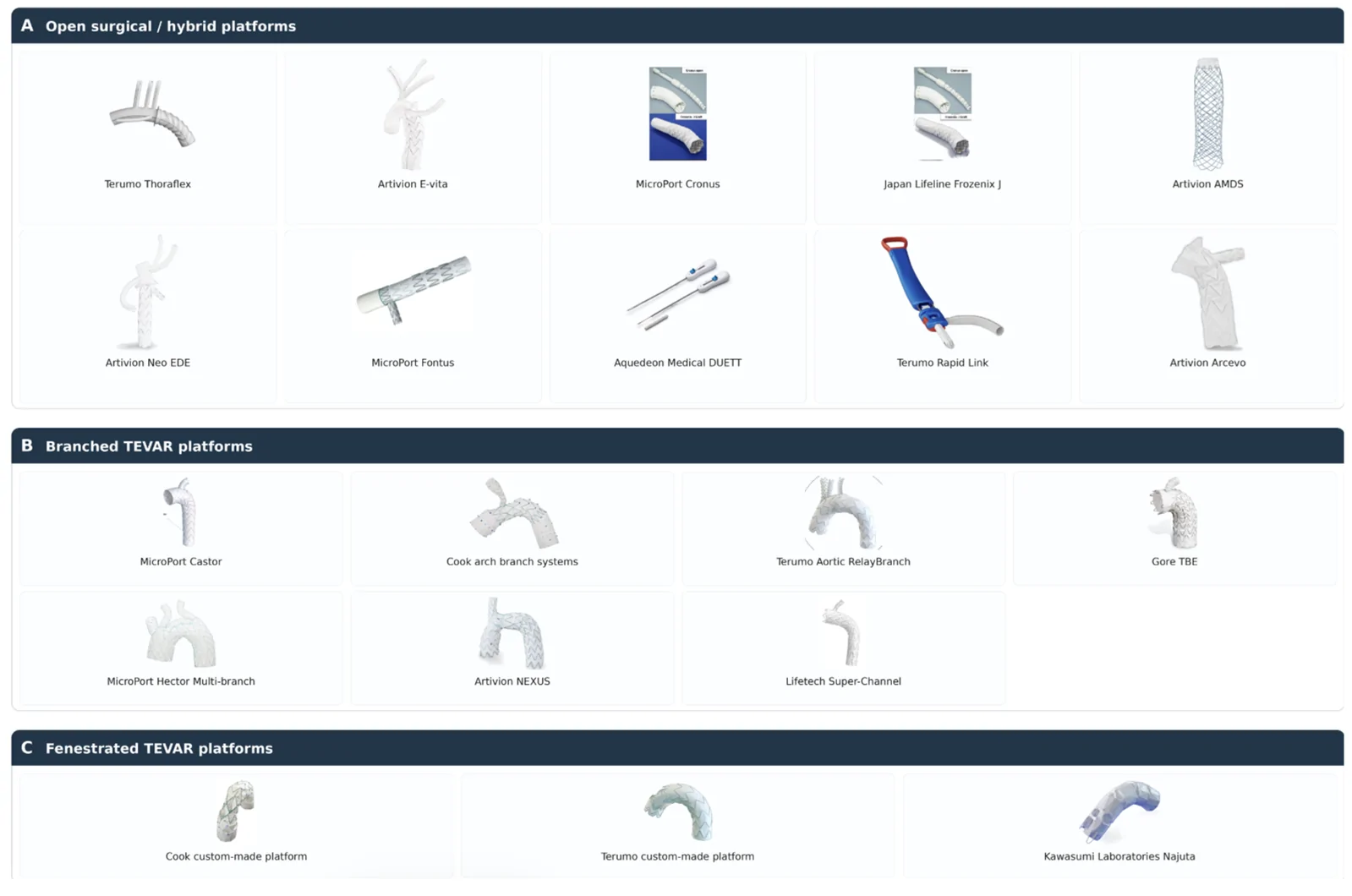
Panel of available solutions for complex aortic arch surgery. (**A**) Open surgical and hybrid solutions; (**B**) B-TEVAR; (**C**) F-TEVAR.

**Figure 2:**
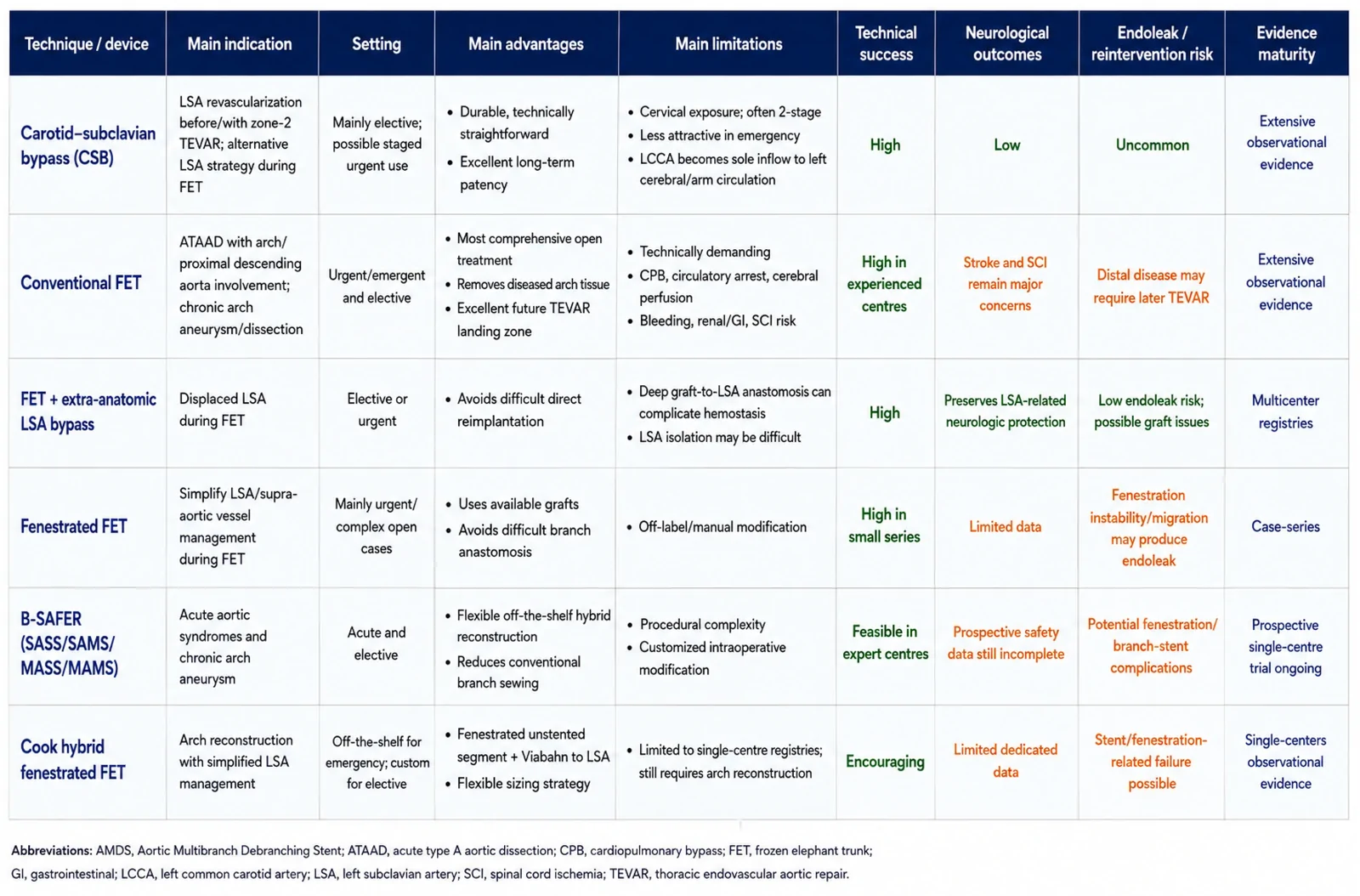
Open arch surgery solutions.

The “debranching first technique” represents another interesting option, allowing more comfortable management of supra-aortic vessels, including the LSA, before circulatory arrest and distal anastomosis [25]. The deep position of the LSA in the chest remains makes the anastomosis technically demanding, time-consuming, and difficult to access in the event of hemostasis problems (Figure 1 and Figure 2).

Strengths

The most comprehensive option for removing diseased aortic tissue (also including dissected supra-aortic vessels), while also providing the ideal landing zone for future endovascular completion with the stented part of the prosthesis.

Limitations

Technically demanding and time-consuming, requiring circulatory lower body arrest with cerebral perfusion with mild-to-deep hypothermia (due to center policies) with related complications.

### 4.3. Thoracoabdominal Open Surgery

In selected cases of aneurysms or dissections, usually chronic (including both Stanford type A and type B disease) involving the thoracic and abdominal aorta, or in cases of failed TEVAR, thoracoabdominal open surgery remains the most invasive but potentially lifesaving bailout solution, primarily for young and fit patients. Depending on the extent of the aortic disease, the Crawford incision (left thoraco-phreno-laparotomy) is usually the preferred approach, which can be extended by a hemiclamshell incision in cases requiring concomitant complete arch surgery. Meticulous attention must be given to the reimplantation of visceral and intercostal arteries. Hypothermia, cerebrospinal fluid drainage, and distal perfusion are employed to protect the spinal cord from ischemia. Even in the most experienced centers, operative mortality ranges from 7 to 11%, with cerebrovascular events (stroke 2–5%) and spinal cord injury (2–6%) as significant complications, in addition to respiratory and renal failure, which are reported with a notable incidence [26,27].

Strengths

Most radical solution for extensive aortic disease; bailout procedure after failed TEVAR.

Limitations

Highly invasive, with substantial operative mortality and morbidity, particularly cerebrovascular and spinal cord complications.

## 5. Hybrid Techniques

### 5.1. FET with Fenestration

To simplify the management of supra-aortic vessels, especially the LSA, manual fenestration of FET prostheses has been proposed. This can be performed using either scissors or electrocautery, creating a fenestration proportional to the vessel ostium. Once positioned in the aortic arch, the fenestration can be secured with fixation sutures, sometimes reinforced with a U-shaped piece of a 4-branched graft and a 4-0 polypropylene running suture [28], or by deploying stents to stabilize the prosthesis and prevent migration and dislodgement, which is particularly important to avoid endoleaks [29,30].

The B-SAFER technique, introduced by Cleveland Clinic in 2009, involves an off-the-shelf tailored stent-graft modification, typically executed in one of four iterations. In the single anastomosis, single stent arch repair technique (SASS), all arch branches remain within the native arch, with the surgical graft sutured to the combined arch tissue and stent-graft, and a single stent placed in the most distal branch, usually the left subclavian artery. The single anastomosis, multiple stents arch repair technique (SAMS) employs stents for multiple branch vessels.

In the multiple anastomoses single stent (MASS) and multiple anastomoses multiple stents (MAMS) techniques, the arch is typically transected in zone 1 or 2, and some arch branches are connected to the prosthetic aortic reconstruction via direct suturing or single/multiple stents. The first two iterations suit acute aortic syndromes, while the latter targets chronic aneurysms.

Device components include a commercially available aortic stent-graft placed in the descending aorta and distal arch, modified by fenestrating the fabric at one or more arch branch orifices, one or more medium branch arch covered stents (Viabahn; WL Gore Fluency or Covera; BD) deployed through these fenestrations into branch vessels, and a conventional surgical graft sutured directly to the stent-grafts. Its effectiveness is currently under investigation in a prospective, single-center, non-blind, non-randomized safety and feasibility study (ClinicalTrials.gov ID NCT04747626) [31].

More recently, in situ laser fenestration has been proposed as an alternative to manual fenestration. This technique requires a 6-Fr sheath advanced from the left brachial artery to reach the aortic stent graft previously implanted. A laser fiber, together with a balloon and Y-valve system, is then advanced through the sheath. Fenestration is then performed and dilated with the balloon; the procedure is completed and “stabilized” with a stent deployment. Given its recent introduction, evidence remains limited in terms of patient numbers and follow-up duration; however, early results are encouraging for LSA reconstruction success and reduction in complications without affecting survival [32].

Another way to obtain a fenestration in FET prosthesis is the electrified-wire technique. From the right common femoral artery, a tulip snare is advanced into the aortic arch. A 0.014′ Astato XS20 wire (Asahi Intecc, Tokyo, Japan) is passed across the ligation point of the LSA, followed by a catheter and a sheath. The Astato wire’s polytetrafluoroethylene (PTFE) is stripped distally and connected to the electrocautery (70 watts; cutting mode), performing the fenestration. Balloon-dilation of the fenestration and stent deployment are the final steps. This technique has thus far been reported only in clinical series and will require further validation [33].

Following the fenestrated FET concept, the Cook hybrid stent graft (Cook, Bloomington, IL, USA) was tailored. This device consists of a distal covered nitinol stent graft and a proximal unstented polyester tubular portion that facilitates the arch anastomosis using conventional arch grafts. It is available in standard, off-the-shelf sizes for emergent cases [in third case, sizes of stented and unstented portions are the same] and can also be customized for elective cases. The device is mounted on a sheath and advanced antegradely; the distal anastomosis is created by suturing the unstented portion with the native aorta and the Dacron graft selected (usually tri- or quadrifurcated). To facilitate LSA anastomosis, before the arch anastomosis, the unstented graft portion was perforated with an electrocautery and a Viabahn stent (W.L. Gore & Assoc, Newark, DE, USA) was deployed in the left subclavian artery [34]. Results with this device are encouraging, but still limited to single-center registries. Cook has now proposed a pre-made fenestrated FET prosthesis to streamline the procedure.

Terumo is now launching two devices, based on the concept of fenestrated FET. Terumo Fenestra (Terumo Aortic, Sunrise, FL, USA) is an innovative device that includes a fenestration on the custom-made platform, with the objective of resolving the LSA anastomosis with a fenestration in the stented portion. Terumo Strada (Terumo Aortic, Sunrise, FL, USA) represents another novel prosthesis characterized by an endobranch emerging from the fenestration, designed to reduce the risk of endoleak (Figure 1 and Figure 2).

### 5.2. Hybrid Stented Branch Devices

Recently, new devices incorporating a stented branch for the LSA have been proposed.

Artivion Neo-EDE (Artivion, Kennesaw, GA, USA) consists of a 50 mm length polyester surgical graft and a 150 mm stented graft. Two branches (available in different sizes) arise from the surgical graft for the brachiocephalic trunk (BCT) and the LCCA. The unique feature is a stented side branch originating from the stented LSA graft, available in 11 mm and 15 mm diameters. The use of the stented branch eliminates the need for extra-anatomical bypass, thereby streamlining the FET procedure. The stented branch is deployed over a guidewire, advanced retrogradely, under direct vision from the LSA ostium or antegradely from the radial or axillary artery. Actually, only the first cases in Europe have been published, with a European registry in construction [35].

The Fontus branch-type intraoperative stent (MicroPort Endovascular MedTech, Shanghai, China), launched in 2021, consists of a nitinol covered stent and a polyester graft with a peculiar stented branch, ideal for LSA. Fontus facilitates total arch repair by shifting the distal anastomosis proximally to Zone 1, thereby simplifying surgical exposure and reducing circulatory arrest time. Additionally, it eliminates the need for LSA mobilization and anastomosis, which significantly reduces overall surgical complexity [36]. In an analysis of 267 consecutive patients, compared with the Cronus stent, the Fontus stent significantly reduced operative time and was not associated with increased postoperative complication rates compared with the traditional Cronus stent [37]. Due to its recent introduction, short-term outcomes of retrospective registries will require confirmation during longer follow-up [38].

**Figure 3:**
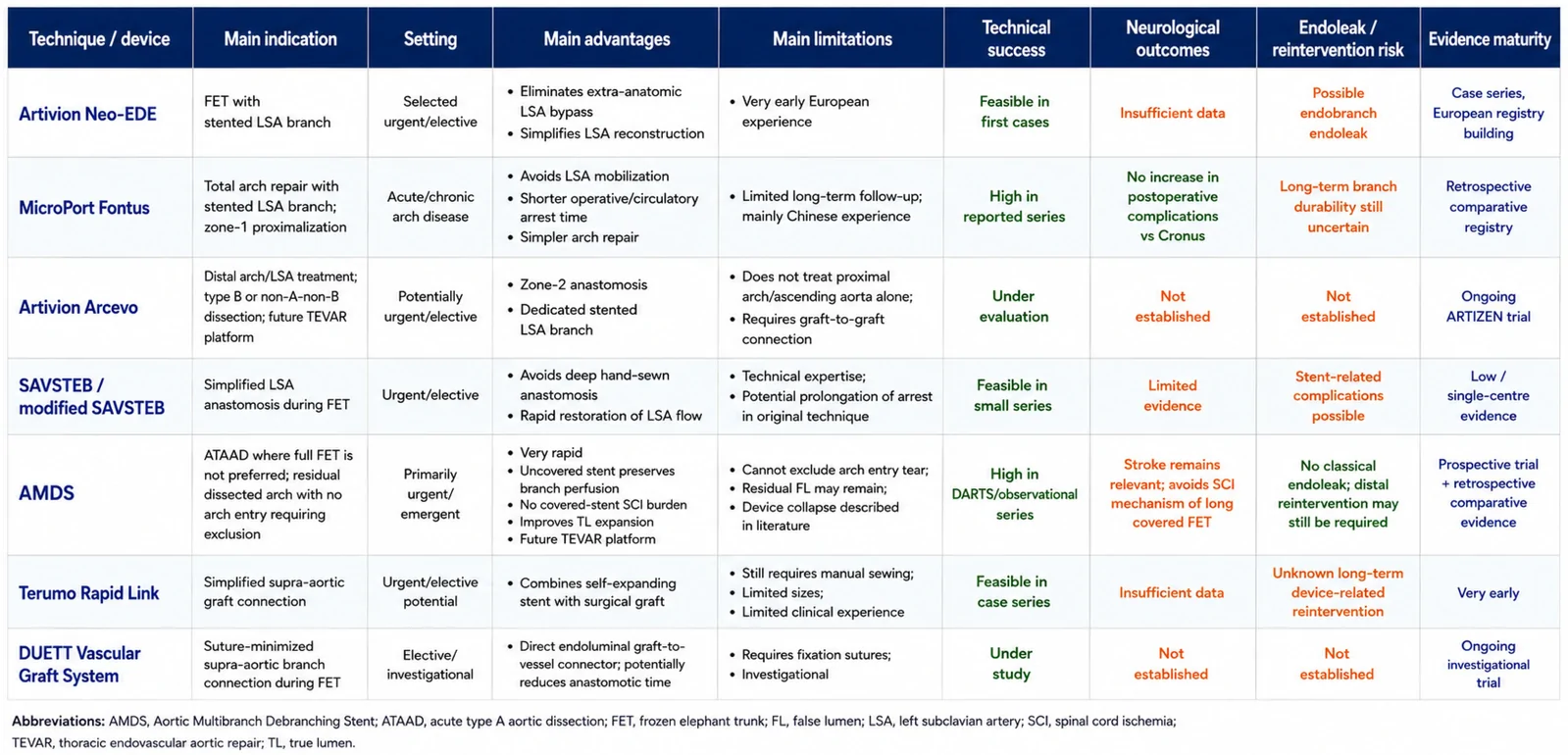
Hybrid techniques and devices.

Arcevo (Artivion, Kennesaw, GA, USA) is another innovative hybrid arch device, designed with a stented branch for LSA, for anastomosis in zone 2. It is under evaluation in pivotal Artizen trial (NCT07089576). Although promising, this solution treats only the distal arch and LSA, requiring an adjunctive prosthesis-to-prosthesis anastomosis for the treatment of proximal aortic arch and ascending aorta. It may represent a useful option in type B or non-A–non-B dissections, while providing an excellent landing zone for future TEVAR (Figure 3).

### 5.3. Supra-Aortic Vessels Bridging Stent Techniques

To facilitate the anastomosis of supra-aortic vessels, especially LSA, new hybrid techniques have emerged. Pichlmaier et al. introduced the Supra-Aortic Vessel anastomosis STEnt Bridging (SAVSTEB), which involves aligning LSA and vascular branch of FET with two to four 4-0 polypropylene sutures and then deploying a stent graft armed with a J-shaped guide wire, introduced under direct vision using an angled clamp through the main body graft [39].

To overcome a potential limitation of this technique, prolonged circulatory arrest during stent deployment, a modified SAVSTEB technique was proposed.

A vascular branch of FET prosthesis is prepared with a U-stitch purse-string a few millimeters distal to the proximal clamp, then a small cut is performed and a stent (in this case Viabahn self-expandable stent (W. L. Gore & Associates, Flagstaff, AZ, USA)) is inserted and advanced into mid-LSA, pre-marked to avoid vertebral artery compromise. After securing the purse-string and tying down the U-stitches, LSA perfusion is restored without bleeding [40]. Single-center experiences represent the only evidence available with mSAVSTEB techniques and will require confirmation in larger studies (Figure 1 and Figure 3).

### 5.4. Hybrid Emerging Devices

AMDS (Artivion, Kennesaw, GA, USA) may have a useful role in cases where LSA cannot be managed surgically and a full FET is not preferred for acute aortic dissection. It consists of an open self-expandable nitinol stent without a surgical graft and has only a proximal sewing collar. It is designed to stabilize the true lumen, reducing pressure and blood flow to the false lumen, with the aim of preventing new distal entry-tear and favoring aortic remodeling. It is available in four sizes. In frail patients with comorbidities who are not ideal candidates for FET, it can provide a good platform even for future TEVAR, without the spinal cord ischemia risk associated with a covered stent graft, although it cannot exclude the false lumen when an entry tear is located in the arch. It can be deployed from zone 0 to zone 2 [41]. Apart from the feasibility study (DARTS trial), evidence is dominated by prospective single-arm data plus retrospective comparative series, PERSEVERE trial will probably add relevant data [41,42].

A new device, Rapid Link (Terumo Aortic, Glasgow, UK), combines a self-expandable stent with a surgical graft that is sutured to the arch graft [43]. Although promising, it still requires manual sewing, limiting time savings. Its full potential lies in integration into hybrid FET prostheses without additional suturing, a capability that will require broader size options. Recent introduction of this device limits results to case series.

The DUETT Vascular Graft System (Aquedeon Medical, Sunnyvale, CA, USA) represents another promising option for simplified supra-aortic anastomoses in FET. This device is a self-expanding covered connector based on an endoluminal, open surgical deployment of a nitinol-reinforced polytetrafluorethylene connection. It is inserted through a small incision in the Dacron portion of the FET device, advanced through 1 of the side branches and positioned with the distal end of the Duett device 2 cm into the target head vessel. Then the deployment of the device creates an anastomosis by directly connecting the surgical graft and target head vessel. The anastomosis is then secured by placing two 4-0 polypropylene stay sutures [44]. Its delivery through the arch-graft side-branch is currently under investigation (NCT06253143) and, if approved by the Food and Drug Administration, it may become a valuable on-label alternative (Figure 1 and Figure 3).

## 6. Endovascular Aortic Surgery/TEVAR

One of the initial reports for stent grafting of thoracic aortic pathology was published in 1994 and interest in the procedure has steadily increased in the procedure [45]. Initial indications for TEVAR were for aneurysmal disease but were soon expanded to treat type B aortic dissections and blunt thoracic aortic injuries (BTAIs). TEVAR has assumed an established role for the majority of aortic pathologies [46,47]. Type B aortic dissection, BTAI, and aneurysmal disease all frequently require TEVAR positioning in zone 2 of the aortic arch. However, as discussed previously, covering the LSA has implications that extend far beyond than simply reducing the blood flow to the left arm.

### 6.1. TEVAR with Chimney Technique

**Figure 4:**
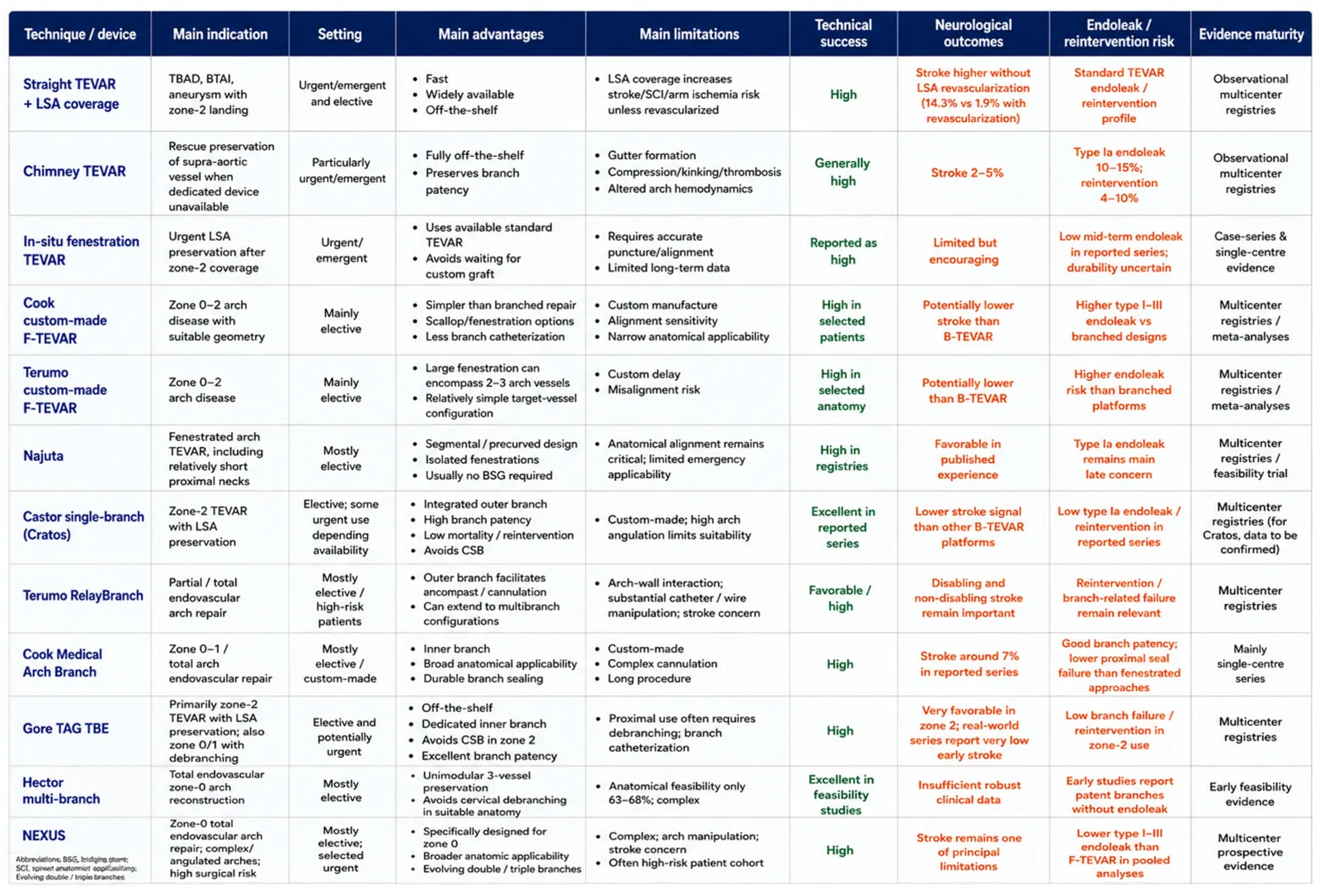
TEVAR techniques and devices.

Particularly useful in time-sensitive settings, the ‘chimney graft’ technique involves placing stents in the side branches of the aorta alongside the main endovascular stent graft. During the procedure, the main aortic endograft is advanced from the femoral artery, while covered stents are advanced from the target supra-aortic vessels, with their proximal ends protruding into the arch [48]. The use of off-the-shelf thoracic stent-grafts and standard covered stents is its main advantage, especially in urgent or emergent settings when custom fenestrated or branched devices are unavailable. Published series report low 30-day mortality (1–5%), good chimney patency, and reasonable mid-term survival in high-risk cohorts [49]. The parallel configuration creates “gutters” between the main graft and chimney stents, predisposing to type Ia endoleaks (around 10–15% on early follow-up). Risks include chimney stent compression, migration, kinking, or thrombosis, as well as stroke (2–5%) and retrograde type A dissection (1–2%). Finally, secondary interventions for endoleaks or chimney-related issues are not negligible (4–10%) [50]. Currently, this technique is reserved only as a rescue intervention when branched and fenestrated TEVAR are unavailable (Figure 4).

Strengths

Availability in time-sensitive settings. Preserves supra-aortic vessel patency.

Limitations

Alters the anatomy and hemodynamics of the aortic arch. Relatively high risk of endoleak and reintervention.

### 6.2. TEVAR with Fenestration In Situ

The challenge of covering the LSA urgently with a straight TEVAR prosthesis, along with its associated risks, can be addressed by adopting in situ fenestration (ISF). The preferred entry site for this procedure is the left brachial or axillary artery. ISF is particularly valuable in urgent or emergent settings when waiting for custom or semi-custom prostheses (e.g., Najuta, Cratos) is not feasible. Several ISF techniques have been described. Early and midterm outcomes [from case series and single-center experiences] support the effectiveness of an adjustable puncture device. After stent graft deployment, an appropriate sheath (often Fustar, Lifetech Scientific Inc., Shenzhen, China) is advanced via brachial or common carotid cutdown perpendicular to the greater curvature of the aortic arch. Once stabilized, fenestration is created, and a V-18 wire (Boston Scientific, Marlborough, MA, USA) is advanced into the ascending aorta through the puncture needle. The fenestration is balloon-dilated sequentially, and a slightly oversized bridging stent is deployed. High technical success and low endoleak rates at midterm follow-up support the efficacy of this approach [51].

An alternative is in situ laser fenestration, performed via the axillary approach using over-the-wire laser systems such as VELAS (GIGAA Laser, Wuhan, China) or the CVX-300 excimer laser system with Turbo-Elite laser atherectomy catheter (Spectranetics, Colorado Springs, CO, USA). Fenestration is balloon-dilated, and a bridging stent is deployed. An increasing number of case series and encouraging early- to midterm results support the feasibility of this technique [52].

Electrosurgical techniques are increasingly being applied in aortic interventions. For LSA in situ fenestrations on thoracic endografts, a sheath is advanced from left brachial arterial access to the LSA ostium adjacent to the endograft. A snare is placed inside the endograft via femoral access, opposing the sheath. A modified Astato wire with activated electrocautery is caught by the snare, creating stable through-and-through access. Balloon dilation and bridging stent deployment complete the procedure. This method, similar in devices and concept, is also used for dissection flap fenestration. Although experience with electrified ISF remains limited, early results from case series are promising [53] (Figure 4).

Strengths

Overcomes limitations of straight TEVAR covering the LSA. Feasible in urgent and emergent settings.

Limitations

Limited patient numbers and mid-to-long-term follow-up data.

### 6.3. Fenestrated TEVAR (F-TEVAR)

F-TEVAR devices represent another valuable alternative, especially when aortic disease involves the inner curvature. Current F-TEVAR designs include proximal landing in Ishimaru zones 0–2 and include various fenestration designs. A notable feature of F-TEVAR is the proximal scallop, where a section of the endograft is removed to face the greater aortic curvature at the proximal end. This design is characteristic of Cook (Bloomington, IA, USA) and Terumo Aortic (Sunrise, FL, USA), which also provides an alternative with a larger fenestration encompassing 2 or 3 aortic arch vessels in a single large cut-out positioned on the greater curvature of the aorta. Najuta endograft (Kawasumi Laboratories, Tokyo, Japan) employs a unique segmental construction with an endoskeleton sheathed in PTFE, favoring isolated fenestrations situated on the outer curvature of the aorta without routinely utilizing bridging stent grafts (BSG). Differently from Terumo and Cook devices, Najuta can also provide a reliable sealing even when proximal neck length is shorter than 25 mm (required by other devices) [54].

Published data help define the advantages and limitations of these devices. A key limitation of F-TEVAR is the potential for misalignment of the scallops or fenestrations with the target vessels after deployment, particularly in the curved anatomy of the aortic arch. Additionally, the long-term behavior of BSGs remains incompletely understood [55]. F-TEVAR is particularly suitable for zone 1–2 disease with relatively straight arch, limited angulation, and predictable supra-aortic origins, where precise fenestration alignment is achievable [56]. A reduced need for extensive catheterization of side branches may translate into slightly lower stroke rates [57]. No side branches mean no branch-stent occlusion risk, at the cost of higher type I–III endoleak rates [55]. Randomized data are limited to foundational Japanese trial, registries and related meta-analyses provide the most consistent results [55,56,57] (Figure 1 and Figure 4).

Strengths

Easier deployment compared with B-TEVAR. Potentially lower stroke rates in selected series. Simpler target-vessel configuration.

Limitations

Risk of intraprocedural and postoperative misalignment, especially in curved arch. Higher endoleak risk. Narrow anatomical applicability. Custom-device delay limits use in urgent/emergent settings. Long-term results are still lacking.

### 6.4. Branched TEVAR (B-TEVAR)

Dedicated branched TEVAR devices have been developed to treat aortic arch disease and supra-aortic vessels. Branches can be classified as inner or outer relative to the main body stent graft. Outer branches, positioned outside the main body, can enhance alignment and provide more space for cannulation but may increase interaction with the arch wall and atheroma [58]. Inner branches are designed to maintain the arch surface as “clean” as possible; they are integrated into the outer curvature wall of the aortic main graft, creating an effective sealing zone for BSG.

Regarding outer-branched TEVAR devices, the Castor Single-Branched Aortic Stent-Graft (MicroPort Endovastec, Shanghai, China) is a custom-made platform designed to treat one supra-aortic vessel, typically the LSA. This device has demonstrated excellent outcomes in terms of technical success, mortality, branch patency, and need for reintervention. Compared to other branched TEVAR devices, it shows lower rates of stroke, type Ia endoleak, and reintervention. However, aortic arches with high angulation remain a significant limitation of this solution [59,60]. The evolution of this graft is the Cratos Single-Branched Aortic Stent-Graft (MicroPort Endovastec, Shanghai, China), recently introduced to the market. Cratos retains the classic bare segment-free integrated branched configuration design, while introducing innovations in its delivery system, significantly enhancing both device performance and surgical experience. A new small-bend posterior release technique combined with Active Birdbeak Control (ABC) improves positioning accuracy and reduces the bird-beak effect. Additionally, the outer sheath has been reduced from 24 F to a 22 F diameter, minimizing vascular access requirements and expanding potential applications.

The Terumo Aortic RelayBranch (Terumo Aortic, Sunrise, FL, USA) features a single outer branch, with the potential for a multi-branch design to treat the aortic arch in favorable anatomies. Reported results are favorable in terms of technical success and early mortality, although disabling and non-disabling strokes remain a concern [61,62].

Conversely, Cook Medical arch branch systems (Cook Medical, Bloomington, IA, USA), custom-made, and the Gore TAG Thoracic Branch Endoprosthesis (TBE) (WL Gore, Flagstaff, AZ, USA) offer an inner-branch design. Cook devices have demonstrated high technical success, low early mortality, acceptable stroke rates (7%), and excellent branch patency at mid-term follow-up. However, evidence remains limited to single-center series [63].

The Gore TBE branch is intended for revascularization of the LSA in zone 2 landings, as specified in its Instructions for Use. Recently, the device has also been employed for the BCT (zone 0) or the LCCA (zone 1), typically in combination with extra-anatomic debranching procedures. This latter approach is particularly relevant in urgent settings. Early results for the Gore TBE are encouraging, with high technical success, low early mortality, and excellent branch patency. Early stroke rates have been reported as 0% in the largest real-world series, with even better outcomes when considering only implantations in zone 2 [64,65].

Beyond the aforementioned devices intended for treating the descending aorta while preserving the LSA, B-TEVAR devices have been developed for the aortic arch and its three supra-aortic vessels. The Hector Multi-branch Thoracic Aortic Stent-graft (MicroPort Endovastec, Shanghai, China) is a unimodular system featuring one outer branch for the BCT, one antegrade inner branch for the LCCA, and one retrograde inner branch for the LSA. Current evidence, though early, is promising, demonstrating high anatomical feasibility (63–68%). While only feasibility studies are available in the literature, results show optimal technical success and patent supra-aortic branches without endoleaks. Larger comparative studies are needed to evaluate stroke, mortality, long-term patency, and reintervention rates [66].

Nexus (Artivion, Kennesaw, GA, USA) is currently the only device in Europe designed for an Ishimaru zone 0 landing. This bimodular device combines arch and ascending aorta components, including one outer branch for the BCT embedded within the arch segment, then modified with a double and triple-branched design, avoiding the need for CSB and CCSB. It is particularly suitable for large-diameter, highly angulated arches, zone 0 involvement, or when the pathology extends across multiple supra-aortic origins. In multicenter comparisons, most F-TEVAR anatomies were also suitable for B-TEVAR, but not vice versa, suggesting that branched devices cover a wider anatomical spectrum [56]. A lower rate of type I–III endoleaks in meta-analyses suggests a more robust seal in challenging arches [57].

Positioned between F-TEVAR and B-TEVAR, the Super-Channel single-branched stent-graft system (Lifetech Scientific, Shenzhen, China) comprises a pre-fenestrated main endograft with a preloaded catheter for LSA cannulation. The device is completed by a specially designed skirt-flared bridging covered stent. This prosthesis is currently being evaluated in a feasibility and safety multicenter prospective clinical trial (NCT06915545), demonstrating excellent technical success in the first 10 cases [67].

Concerning proximal neck length requirements, no branched TEVAR system specifies a formal minimum proximal neck length. Planning therefore defaults to standard TEVAR sealing requirements (20–25 mm), even for devices with an intrinsic structure that favors fixation (Castor single outer branch), with Zone 0–1 devices (Nexus, Hector, Cook triple-branch) often requiring longer sealing zones due to ascending-aorta fixation.

Actually, the literature consists of multicenter prospective registries and related metanalyses [59,68,69] (Figure 1 and Figure 4).

Strenghts

No need for CSB. Single-stage procedure suitable for patients at high surgical risk or with significant comorbidities. Broader anatomical applicability. Lower rates of proximal and distal endoleaks. Growing off-the-shelf options.

Limitations

More complex than F-TEVAR because of branch catheterization, longer fluoroscopy and wire times, and greater arch manipulation. Stroke risk (higher than that of F-TEVAR in some series) [56,68,69]. Branch-related failure.

## 7. Discussion

The nature of the underlying aortic disease strongly influences the optimal therapeutic strategy. Acute type A aortic dissection requires urgent definitive intervention, often serving as a “first-stage” procedure to facilitate subsequent endovascular treatments. Off-the-shelf FET prostheses currently represent the gold standard, with accumulating long-term data underscoring their central role [70,71]. In patients with significant comorbidities unsuitable for FET, ascending aorta replacement with concomitant proximalization of at least 2 supra-aortic vessels may serve as a strategic landing zone for a staged TEVAR in zone 0. Emerging hybrid arch devices present promising alternatives by reducing operative time and technical demands.

In urgent scenarios involving type B and non-A–non-B dissections, FET remains a compelling option, especially when the dissection extends near the LSA, necessitating coverage during TEVAR. A staged approach with initial FET followed by TEVAR may offer a personalized and durable solution, recognizing aortic disease as a chronic, lifelong condition. For frail patients, CSB followed by straight TEVAR may be preferable. The increasing availability of off-the-shelf B-TEVAR devices, particularly those preserving LSA flow, expands treatment possibilities, provided that anatomical criteria such as adequate collar length between LSA and LCCA are met. Multi-branched TEVAR remains less accessible in urgent settings due to its custom-made nature, and the current outcomes remain limited by a significant number of cerebrovascular events.

**Figure 5:**
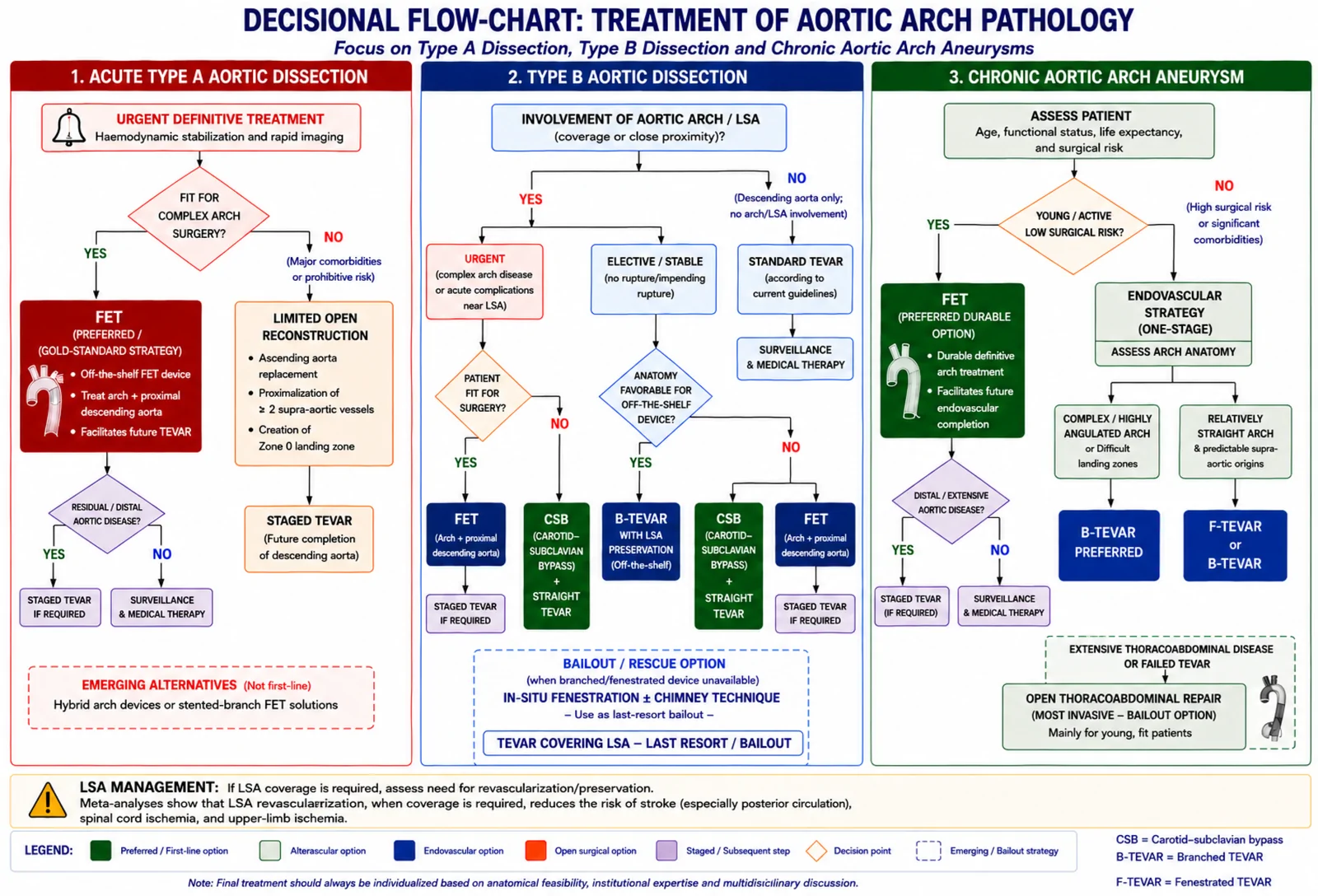
Flow-chart for treatment of aortic arch pathology, with involvement of LSA.

For chronic arch aneurysms, all of the aforementioned modalities remain viable. Patient functional status and age often guide treatment selection; younger, active patients may benefit from FET given its durable outcomes, whereas high-risk patients may be better suited to one-stage procedures favoring B-TEVAR over F-TEVAR, even in anatomically complex aortic arches (Figure 5).

## 8. Conclusions

Management of the LSA during aortic arch surgery remains a pivotal and intricate component of treatment, with a major impact on patient outcomes. Revascularization of the LSA is essential in selected clinical settings to safeguard cerebral, spinal cord, and upper limb perfusion, thereby markedly diminishing the risks of stroke, spinal cord injury, and ischemic complications. The FET technique has transformed open aortic arch surgery by providing a comprehensive and effective approach with promising long-term outcomes, despite its technical complexity and associated risks.

Emerging hybrid and endovascular strategies, including branched and fenestrated TEVAR alongside innovative stented branch devices, offer valuable, patient-tailored alternatives. These modalities aim to reduce operative duration, minimize invasiveness, and broaden therapeutic options, and may particularly benefit high-risk or comorbid patients.

Ultimately, the selection of surgical or endovascular intervention must be individualized, taking into account the specific aortic pathology, urgency of treatment, patient comorbidities, and anatomical considerations. A patient-centric, staged approach that integrates open and endovascular techniques may optimize outcomes in complex aortic arch disease, exemplifying the evolving paradigm of aortic surgery where innovation and precision converge to enhance patient care.

## Data Availability

All data are available on request from the corresponding author.

## Abbreviations

The following abbreviations are used in this manuscript:

LSA	Left subclavian artery
FET	Frozen elephant trunk
TEVAR	Thoracic endovascular repair
CSB	Carotid-to-subclavian bypass
BCT	Brachiocephalic trunk
LCCA	Left common carotid artery
